# Nanofat promotes wound healing in skin following exposure to ionizing radiation

**DOI:** 10.1038/s41598-025-17961-8

**Published:** 2025-08-29

**Authors:** Ettore Limido, Andrea Weinzierl, Emmanuel Ampofo, Claudia E. Rübe, Gargi Tewary, Yves Harder, Michael D. Menger, Matthias W. Laschke

**Affiliations:** 1 Institute for Clinical and Experimental Surgery, Saarland University, PharmaScienceHub (PSH), 66421 Homburg, Germany; 2 Department of Plastic Surgery and Hand Surgery, University Hospital Zurich, Zurich, 8006 Switzerland; 3 Department of Radiation Oncology, Saarland University, 66421 Homburg, Germany; 4 Department of Plastic, Reconstructive and Aesthetic Surgery and Hand Surgery, Centre Hospitalier Universitaire Vaudois (CHUV), Lausanne, 1011 Switzerland; 5 Faculty of Biology and Medicine, University of Lausanne (UNIL), Lausanne, 1005 Switzerland

**Keywords:** Wound healing, Radiotherapy, Nanofat, Platelet-rich plasma, Vascularization, Dorsal skinfold chamber, Diseases, Medical research

## Abstract

Radiotherapy, while effective in cancer treatment, can lead to side effects, such as radiodermatitis with potential long-term consequences including telangiectasias, ulceration and fibrosis of the skin, eventually resulting in impaired wound healing. In this study, we analyzed whether the healing of such challenging wounds can be improved by nanofat (NF). NF is generated by mechanical emulsification and filtration of fat samples and, thus, is a random mixture of adipose-derived stem cells, microvascular fragments, extracellular matrix components and growth factors. Two months after localized ionizing radiation of the skin with a total dose of 20 Gy, full-thickness wounds were created in dorsal skinfold chambers of mice, which were filled with platelet-rich plasma (PRP; control, *n* = 8) or NF fixed in PRP (PRP + NF, *n* = 8). The healing process was assessed by means of stereomicroscopy, intravital fluorescence microscopy, histology and immunohistochemistry over 14 days. The closure of PRP + NF-treated wounds was accelerated, as indicated by significantly smaller wound areas on day 14 when compared to controls. This was associated with a higher density of blood-perfused microvessels inside the wounds. Moreover, PRP + NF-treated wounds showed a tendency towards an improved granulation tissue formation, lymphatic drainage and M2/M1 macrophage ratio. Taken together, these findings suggest that the application of NF represents a promising therapeutic strategy for the management of complex wounds in irradiated skin.

## Introduction

The skin is an essential multi-functional organ acting as a mechanical barrier and sensitive organ as well as participating at thermoregulation and immune defense against external pathogens^1^. Disruption of this barrier triggers the wound healing cascade, which progresses through the overlapping phases of hemostasis/inflammation, proliferation and remodeling, ultimately leading to the formation of an avascular scar^2^. However, comorbidities or therapeutic interventions, such as radiotherapy, can significantly impair skin physiology and, thus, also this healing process^3–5^.

Radiotherapy, a cornerstone in cancer treatment, is applied in over 60% of patients undergoing adjuvant or neoadjuvant therapy^6–8^. While ionizing radiation effectively targets tumor cells, it can also damage the healthy skin overlying the treated tumor and cause acute complications, including radiodermatitis, ranging in severity from transient erythema to non-healing ulcers^9–12^. An important hallmark of radiodermatitis is the impairment of the mitotic ability of stem/progenitor cells in the basal cell layers due to radiation-induced DNA damage, leading to suppressed cell renewal in the epidermis. In parallel, radiation induces premature senescence in differentiated keratinocytes and the secretion of pro-inflammatory cytokines and chemokines. These effects lead to chronic skin inflammation and an overall weakening of barrier integrity^12^. Radiodermatitis is also often associated with subsequent tissue alterations, resulting in fibrosis due to inflammatory and pro-fibrotic pathways. These pathways are mainly activated by transforming growth factor (TGF)-β1, resulting in irregular cell proliferation, thrombosis and hypoperfusion^13,14^. This, in turn, can complicate subsequent surgeries, such as those after neoadjuvant cancer treatment or for the management of other radiotherapy-induced complications^15–17^. Moreover, it can markedly affect physiological wound healing and consequently cause non-healing ulcers. Accordingly, there is an urgent clinical need for effective wound management after previous irradiation or in case of challenging healing conditions. The application of nanofat (NF) may represent a promising approach for this clinical challenge.

NF is a fluid autologous fat derivative, which can be rapidly generated by mechanical emulsification (20–30 passes with a connector size between 1.2 and 1.6 mm) and filtration (pore size: ~300–500 μm) of lipoaspirates^18,19^. This leads to the destruction of most mature adipocytes, while other tissue components survive the procedure. Accordingly, NF represents a random mixture of adipose-derived stem cells (ASCs), microvascular fragments, extracellular matrix (ECM) and growth factors. The combination of these components has a high regenerative capacity^19–21^. Recently, we could demonstrate that NF fixed in platelet-rich plasma (PRP) promotes wound healing in healthy skin^21^. In fact, we found that PRP + NF-treated wounds exhibit an improved vascularization, lymphatic drainage and closure when compared to untreated and PRP-treated wounds^21^. In contrast, the healing of untreated and PRP-treated wounds did not markedly differ from each other^21^. Moreover, other studies reported that autologous fat transfer can be used to reverse radiation-induced tissue damage^10,14,22^. For instance, Rigotti and colleagues^10^ demonstrated that repeated low-invasive injections of purified autologous lipoaspirates in breast cancer patients suffering from radiation-induced fibrosis following radiotherapy markedly improve the dermal architecture of the irradiated tissue with increased hydration and neovessel formation. These regenerative effects may be mainly attributed to ASCs, which can differentiate into native cells and secrete various growth factors and cytokines that induce angiogenesis and disrupt pro-fibrotic pathways, such as TGF-β1 signaling^14,22^. However, despite these promising findings, the effects of NF on wound healing in skin following exposure to ionizing radiation have not been analyzed so far.

Therefore, we created full-thickness wounds in dorsal skinfold chambers of mice two months after localized ionizing radiation of the skin with a total dose of 20 Gy to induce radiodermatitis. These wounds were then treated with PRP (control) or NF fixed in PRP (PRP + NF) and their healing was assessed by means of repeated stereomicroscopy and intravital fluorescence microscopy throughout an observation period of 14 days as well as histology and immunohistochemistry at the end of the in vivo experiments.

## Results

### Macroscopic appearance of the skin and body weight development after ionizing radiation

After localized, single-course ionizing radiation of the dorsal skinfold with a total dose of 20 Gy, all mice developed acute radiodermatitis. This was characterized by delayed hair growth and skin alterations ranging from erythema to excoriations with a peak inflammatory response at 2 weeks post-irradiation. Thereafter, the skin and hair follicles continuously regenerated. Moreover, gray fur developed due to irreversible damage of melanocytes within the irradiated skinfold. In addition, the skinfold finally exhibited a higher stiffness and randomly distributed small scars at the time point of the dorsal skinfold chamber preparation.

**Fig. 1:**
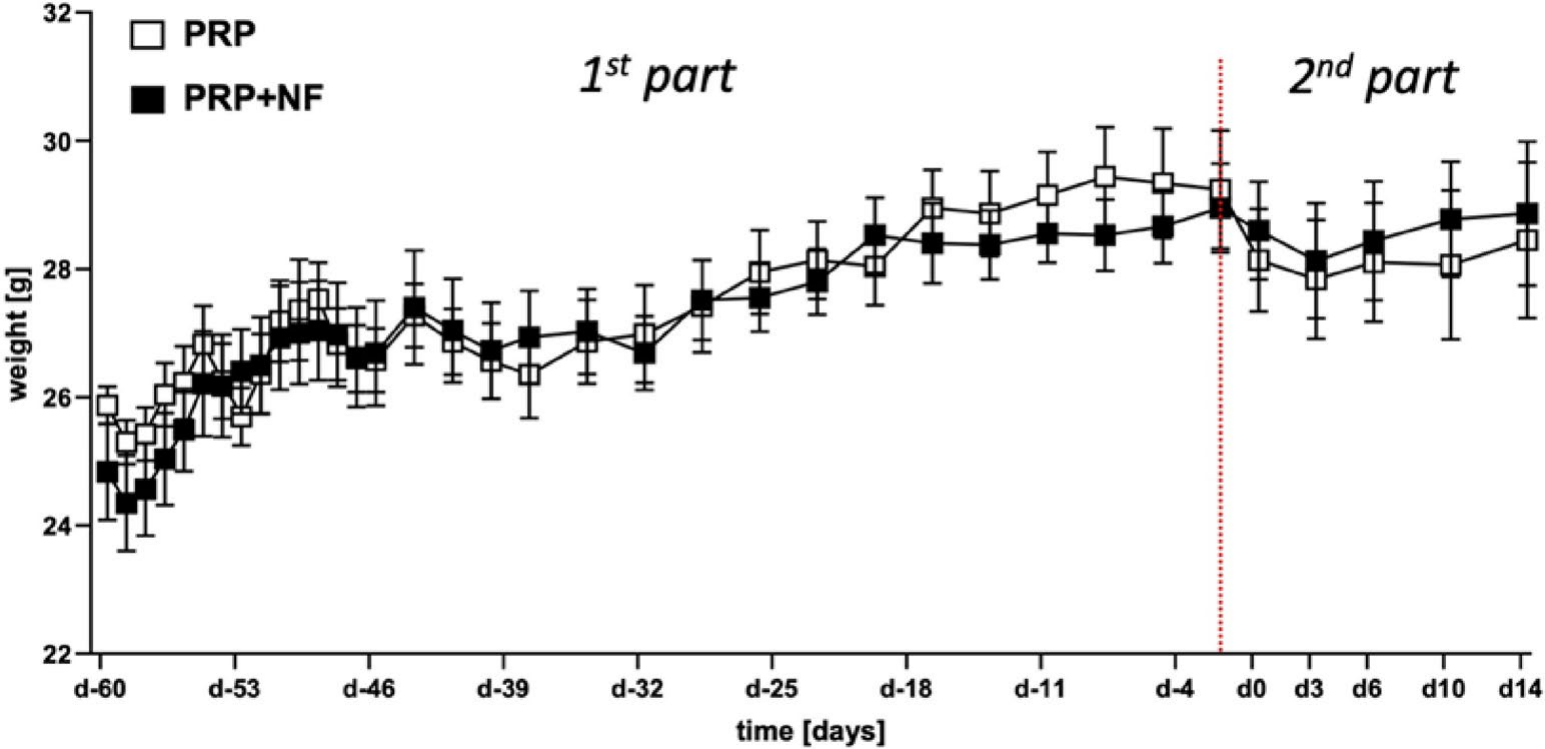
Development of body weight. Body weight (g) of mice that underwent localized ionizing radiation of the dorsal skinfold on day − 60 and were equipped with dorsal skinfold chambers on day − 2 (red dotted line). Within the chambers, wounds were created on day 0, which were then treated with PRP (white squares; *n* = 8) or a combination of PRP and NF (PRP + NF; black squares; *n* = 8). Mean ± SEM.

The body weight of the animals slightly decreased during the first days as well as at the beginning of the second and third week after ionizing radiation (Fig. 1). Throughout the following observation period, the mice progressively gained body weight. A similar course could be observed after the preparation of the dorsal skinfold chamber, which was also associated with a short reduction in body weight during the first 5 days post-surgery (Fig. 1).

### In vivo microscopy of healing wounds

**Fig. 2:**
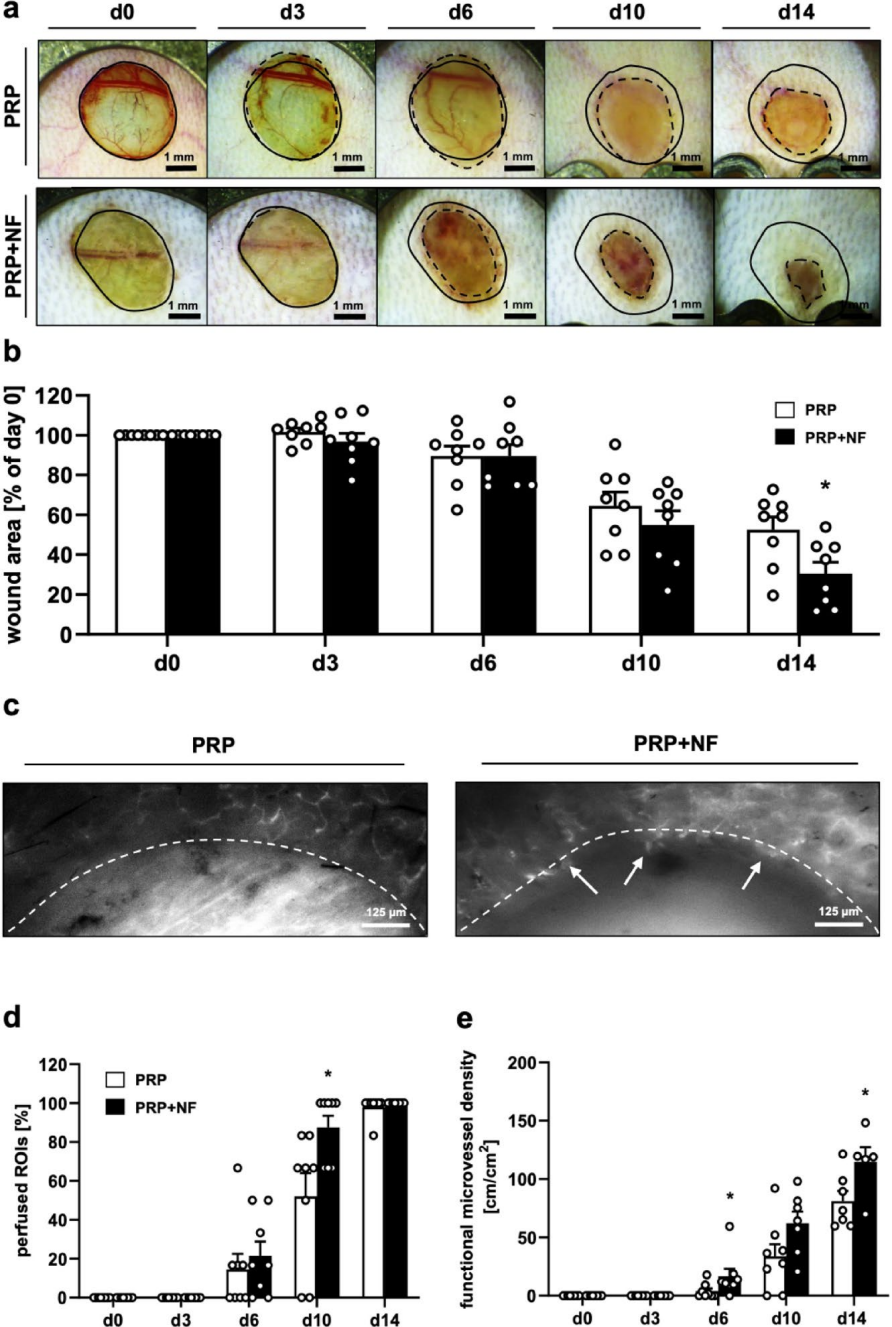
In vivo microscopy of healing wounds within dorsal skinfold chambers. (**a**) Stereomicroscopic images of a PRP-treated and a PRP + NF-treated wound on days 0, 3, 6, 10 and 14 (solid lines = initial wound borders; broken lines = wound borders at the indicated time points). (**b**) Wound area (% of day 0) of PRP-treated (white bars; *n* = 8) and PRP + NF-treated (black bars; *n* = 8) wounds on days 0, 3, 6, 10 and 14, as assessed by stereomicroscopy. Mean ± SEM. *p < 0.05 vs. PRP. (**c**) Intravital fluorescence microscopic images of a PRP-treated and a PRP + NF-treated wound with first blood-perfused microvessels at the wound border (arrows) on day 6 (broken lines = wound borders). (**d**,** e**) Perfused ROIs (d, %) and functional microvessel density (**e**, cm/cm²) of PRP-treated (white bars; *n* = 8) and PRP + NF-treated (black bars; *n* = 8) wounds on days 0, 3, 6, 10 and 14, as assessed by intravital fluorescence microscopy. Mean ± SEM. *p < 0.05 vs. PRP.

The healing of PRP- and PRP + NF-treated wounds within dorsal skinfold chambers was repeatedly analyzed by means of stereomicroscopy and intravital fluorescence microscopy over an observation period of 14 days (Fig. 2). Stereomicroscopic analyses revealed an accelerated healing process in PRP + NF-treated wounds, which exhibited a significantly reduced wound area on day 14 when compared to PRP-treated control wounds (Fig. 2a and b).

First blood-perfused microvessels could be detected in the border zones of the wounds of both groups by means of intravital fluorescence microscopy on day 6 (Fig. 2c and d). Throughout the following days, these microvessels progressively grew towards the center of the wounds and branched into new microvascular networks. In line with our stereomicroscopic results, this vascularization process was also accelerated in PRP + NF-treated wounds, as indicated by a significantly increased fraction of perfused regions of interest (ROIs) on day 10 and a significantly higher functional microvessel density on days 6 and 14 when compared to PRP-treated control wounds (Fig. 2d and e). The additional assessment of microhemodynamic parameters revealed no marked differences between the two groups (Table 1). The microvessels within PRP- and PRP + NF-treated wounds showed a decrease of diameters and an increase of centerline red blood cell (RBC) velocity and shear rate over time, which is a known process occurring during maturation and remodeling of newly developing microvascular networks.

**Table 1:** Diameter (µm), centerline RBC velocity (µm/s), shear rate (s^–1^) and volumetric blood flow (pL/s) of microvessels within wounds filled with PRP (*n* = 8) or PRP + NF (*n* = 8) on days 0, 3, 6, 10 and 14, as assessed by intravital fluorescence microscopy. Mean ± SEM.

	d0	d3	d6	d10	d14
Diameter [µm]:					
PRP	–	–	19.6 ± 1.7	18.4 ± 1.0	16.1 ± 0.6
PRP + NF	–	–	18.0 ± 0.4	16.7 ± 0.8	15.5 ± 0.5
Centerline RBC velocity [µm/s]:					
PRP	–	–	69.2 ± 9.8	83.1 ± 5.3	123.9 ± 19.0
PRP + NF	–	–	90.7 ± 13.0	103.3 ± 17.6	112.4 ± 19.4
Shear rate [s^–1^]:					
PRP	–	–	32.4 ± 5.8	39.9 ± 3.9	66.6 ± 11.9
PRP + NF	–	–	40.9 ± 5.8	53.3 ± 10.8	63.7 ± 13.2
Volumetric blood flow [pL/s]:					
PRP	–	–	11.3 ± 1.0	13.7 ± 1.6	15.7 ± 1.8
PRP + NF	–	–	16.2 ± 2.8	13.7 ± 1.8	12.5 ± 1.5

### Histological and immunohistochemical analysis of the wounds

**Fig. 3:**
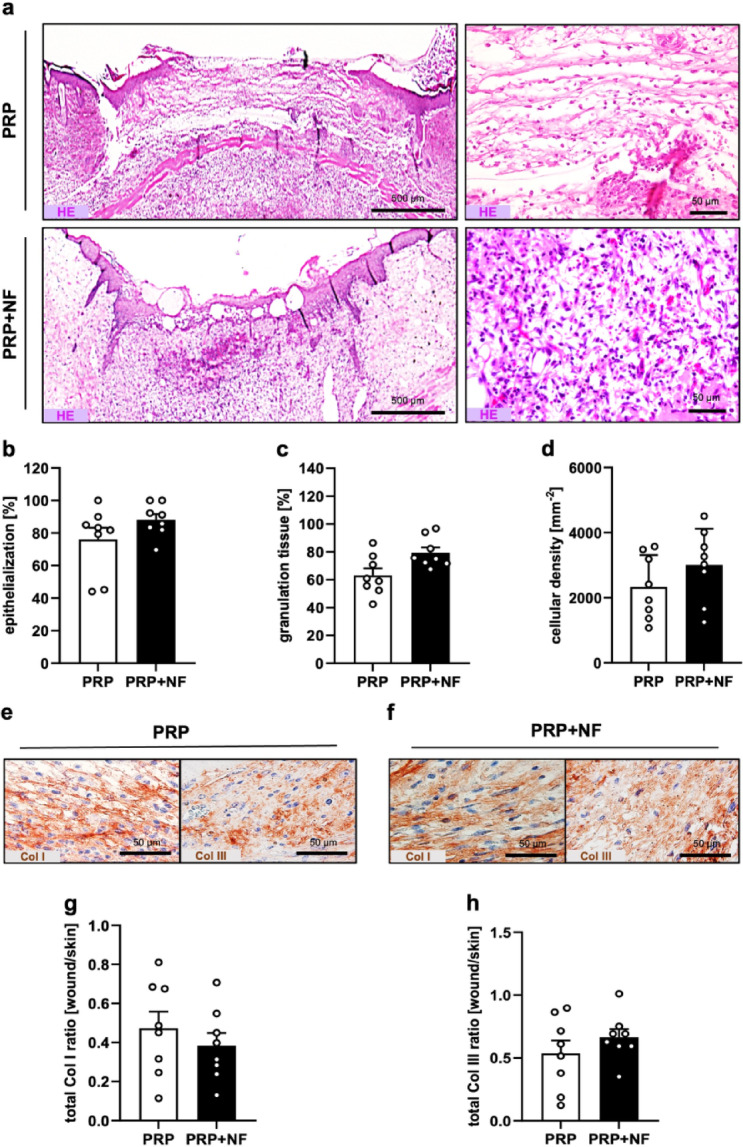
Tissue formation in healing wounds. (**a**) HE-stained sections (left panels = overview of the wounds showing their epithelialization; right panels = higher magnification of the granulation tissue in the wound center) of a PRP-treated and a PRP + NF-treated wound on day 14. (**b-d**) Epithelialization (**b**, %), granulation tissue formation (**c**, %) and cellular density (**d**, cells/mm^− 2^) of PRP-treated (white bars; *n* = 8) and PRP + NF-treated (black bars; *n* = 8) wounds on day 14, as assessed by histology. Mean ± SEM. (**e**,** f**) Immunohistochemical detection of Col I and Col III in a PRP-treated and a PRP + NF-treated wound on day 14. (**g**,** h**) Total Col I (**g**) and Col III (**h**) ratio (wound/normal skin) of PRP-treated (white bars; *n* = 8) and PRP + NF-treated (black bars; *n* = 8) wounds on day 14, as assessed by immunohistochemistry. Mean ± SEM.

At the end of the in vivo experiments, the wounds were analyzed by means of histology and immunohistochemistry. The analysis of hematoxylin-eosin (HE)-stained sections demonstrated that PRP + NF-treated wounds showed a tendency towards a higher epithelialization, granulation tissue formation and cellular density when compared to PRP-treated control wounds (Fig. 3a-d). This, however, was not statistically significant. Additional immunohistochemical analyses revealed a similar total collagen (Col) I and Col III ratio in the wounds of both groups (Fig. 3e-h).

**Fig. 4:**
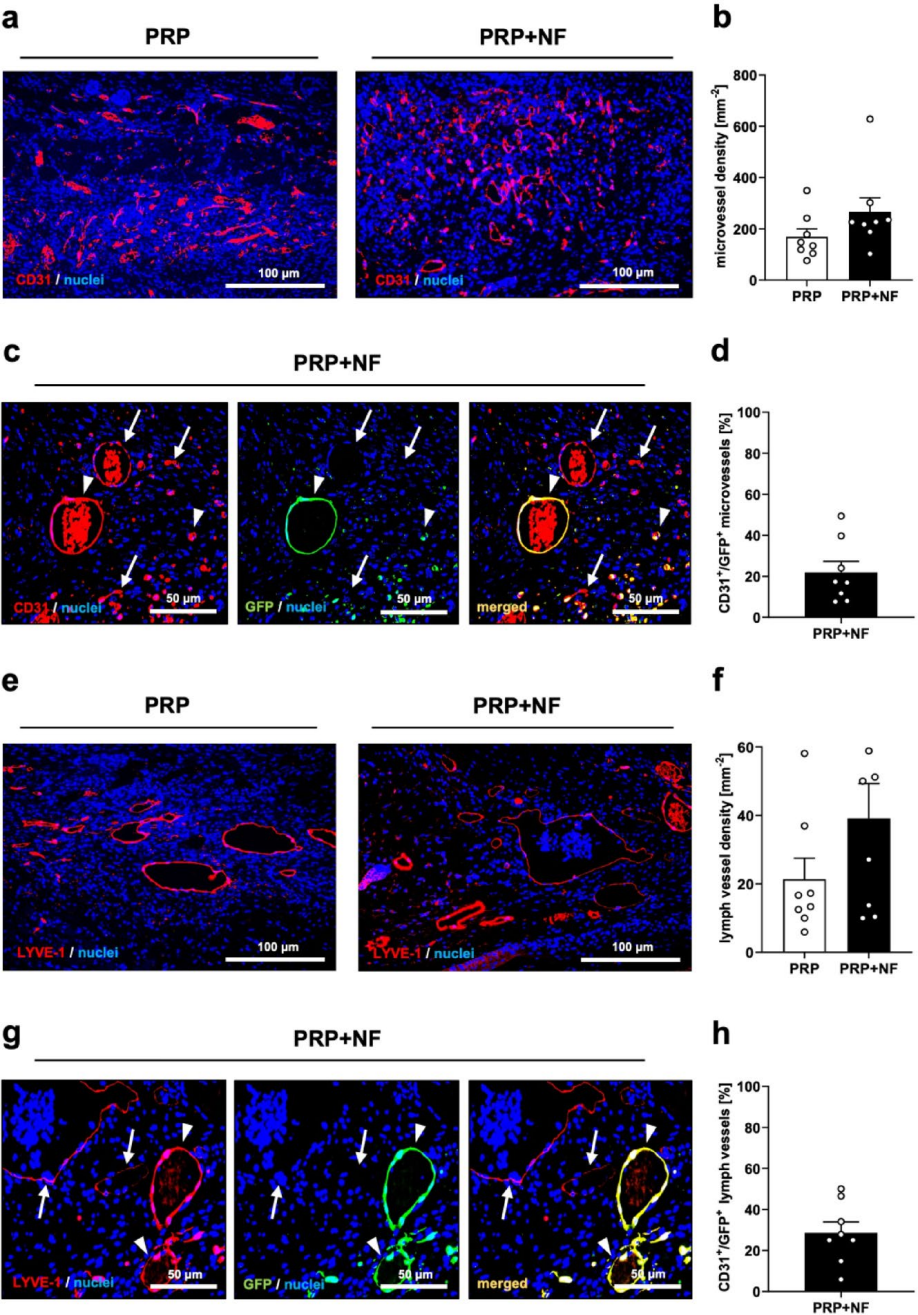
Vascularization and lymphatic drainage of healing wounds. (**a**) Immunohistochemical detection of CD31^+^ microvessels in a PRP-treated and a PRP + NF-treated wound on day 14. (**b**) Microvessel density (mm^− 2^) of PRP-treated (white bars; *n* = 8) and PRP + NF-treated (black bars; *n* = 8) wounds on day 14, as assessed by immunohistochemistry. Mean ± SEM. (**c**) Immunohistochemical detection of CD31^+^/GFP^−^ (arrows) and CD31^+^/GFP^+^ (arrowheads) microvessels in a PRP + NF-treated wound on day 14. (**d**) CD31^+^/GFP^+^ microvessels (%) in PRP + NF-treated wounds (black bar; *n* = 8) on day 14, as assessed by immunohistochemistry. Mean ± SEM. (**e**) Immunohistochemical detection of LYVE-1^+^ lymph vessels in a PRP-treated and a PRP + NF-treated wound on day 14. (**f**) Lymph vessel density (mm^− 2^) of PRP-treated (white bars; *n* = 8) and PRP + NF-treated (black bars; *n* = 8) wounds on day 14, as assessed by immunohistochemistry. Mean ± SEM. (**g**) Immunohistochemical detection of LYVE-1^+^/GFP^−^ (arrows) and LYVE-1^+^/GFP^+^ (arrowheads) lymph vessels in a PRP + NF-treated wound on day 14. (H) LYVE-1^+^/GFP^+^ lymph vessels (%) in PRP + NF-treated wounds (black bar; *n* = 8) on day 14, as assessed by immunohistochemistry. Mean ± SEM.

By means of CD31 and lymphatic vessel endothelial hyaluronan receptor (LYVE)-1 stainings, we found a not statistically significant trend towards a higher density of microvessels and lymph vessels in PRP + NF-treated wounds (Fig. 4a, b, e and f). CD31/green fluorescent protein (GFP) and LYVE-1/GFP co-stainings further revealed that ~ 20% of microvessels and ~ 30% of lymph vessels within PRP + NF-treated wounds exhibited a GFP signal, indicating their origin from the GFP^+^ vessels of the NF (Fig. 4c, d, g and h).

**Fig. 5:**
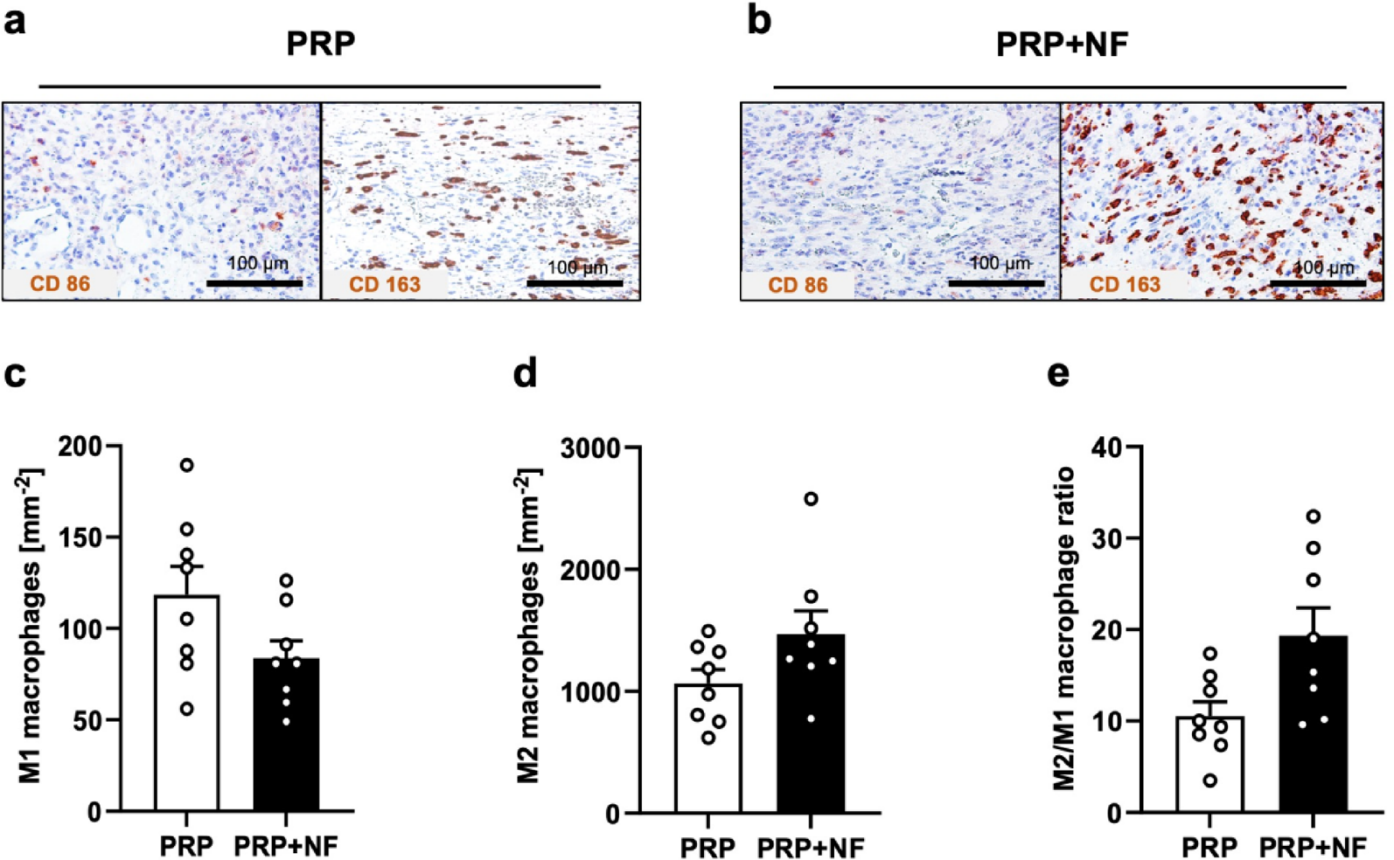
Macrophage infiltration of healing wounds. (**a**,** b**) Immunohistochemical detection of CD86^+^ M1 and CD163^+^ M2 macrophages in a PRP-treated and a PRP + NF-treated wound on day 14. (**c-e**) M1 macrophages (**c**, mm^− 2^), M2 macrophages (**d**, mm^− 2^), and M2/M1 macrophage ratio (**e**) in PRP-treated (white bars; *n* = 8) and PRP + NF-treated (black bars; *n* = 8) wounds on day 14, as assessed by immunohistochemistry. Mean ± SEM.

Finally, we assessed the amount of M1 and M2 macrophages in the wounds by means of CD86 and CD163 stainings (Fig. 5a and b). PRP + NF-treated wounds showed a not statistically significant tendency towards a lower number of M1 macrophages and a higher number of M2 macrophages, resulting in a higher M2/M1 macrophage ratio when compared to PRP-treated control wounds (Fig. 5c-e).

## Discussion

NF is an autologous fat derivative, which is increasingly used in plastic, reconstructive and esthetic surgery for several indications, such as wound management, scar repair, facial rejuvenation and alopecia treatment^14^. This is due to its high regenerative activity because it is a rich source of ASCs, microvascular fragments, ECM components and growth factors^18,19^. Accordingly, we herein could demonstrate that NF effectively promotes wound healing even under challenging healing conditions within irradiated skin.

The treatment of challenging wounds, e.g. following ionizing radiation, can be difficult due to alterations in skin physiology^3,23^. In fact, ionizing radiation damages the skin through directly breaking DNA and generating reactive oxygen species that impair cellular function and activate inflammatory pathways^13^. In line with these mechanisms, we observed the development of acute radiodermatitis of the dorsal skinfold during the initial 1–3 weeks following single exposure of mice to a localized, ionizing radiation with 20 Gy. After two months, this was associated with a higher stiffness of the skin at the time point of dorsal skinfold chamber preparation and wound creation, which may be explained by the dysregulation of ECM production and remodeling, resulting in fibrotic changes^4^. These observations indicate that our experimental approach was suitable to mimic challenging wound healing conditions following radiotherapy in a preclinical setting.

It is well known that ionizing radiation markedly affects tissue perfusion and oxygenation through different mechanisms^10,11,24^. For instance, radiation doses of 2–12 Gy have been shown to inhibit the proliferation of microvascular endothelial cells^25^. Moreover, radiation increases microvascular permeability due to alterations of tight junctions between endothelial cells, which is associated with a higher risk of occlusive arterial diseases^26–28^. This endothelial dysfunction not only impairs blood supply but also compromises immune cell trafficking, further exacerbating the inflammatory response and delaying repair ​^12^. On the other hand, several studies reported the beneficial effects of adipose tissue components on angiogenesis and endothelial cell viability^11,14,29,30^. In addition, we have recently shown that NF contains many angiogenic growth factors and microvascular fragments, which effectively improve the vascularization of target tissues^21,31^. NF is therefore a promising candidate to accelerate wound healing. As an extension of this previous research, we herein found that PRP + NF-treated wounds exhibited an accelerated and improved vascularization, even after the application of ionizing radiation. Thereby, intravital fluorescence microscopy revealed a significantly higher fraction of perfused ROIs and functional microvessel density when compared to PRP-treated control wounds.

In contrast to our intravital fluorescence microscopic findings, we did not detect statistically significant differences in the immunohistochemically assessed vascularization of PRP-treated and PRP + NF-treated wounds, although there was a trend towards a higher density of CD31^+^ microvessels in PRP + NF-treated wounds. It should be considered, however, that the latter analysis technique only measures the number of microvessels per tissue area without assessing their functionality^32^, as it is achieved by the visualization of fluorescein isothiocyanate (FITC)-dextran-labeled blood perfusion during intravital fluorescence microscopy^31^. On the other hand, immunohistochemistry bears the advantage of gaining additional information about the origin of microvessels inside the wounds by CD31/GFP co-stainings. These co-stainings revealed that ~ 20% of the microvessels within PRP + NF-treated wounds directly originated from the NF of the GFP^+^ donor mice. Moreover, we detected ~ 30% GFP^+^ lymph vessels within PRP + NF-treated wounds. This shows that even though the microenvironment of these wounds was altered after ionizing radiation, these vessel components inside the NF were able to survive and to contribute to the formation of new microvascular and lymphatic networks within the wounds, as previously reported for wounds in healthy skin^21^.

Finally, we investigated the infiltration of the wounds with M1 and M2 macrophages. Also not proven significant, we found a trend towards a higher M2/M1 macrophage ratio in PRP + NF-treated wounds when compared to controls. Thus, it may be speculated that NF drives macrophage polarization towards an M2 phenotype, which may have contributed to the accelerated healing of PRP + NF-treated wounds. In fact, M2 macrophages are known to enhance angiogenesis, collagen synthesis and tissue remodeling^33^. In line with our results, previous studies have also shown that adipose-derived products, such as NF, can modulate macrophage polarization and, thus, promote tissue regeneration^34^.

In summary, the present study shows for the first time that the local application of NF is a promising therapeutic strategy for the management of complex wounds in irradiated skin. Nonetheless, it should be considered that this study also has some limitations. For instance, it lacks data on the expression of inflammatory cytokines or molecular markers for the assessment of potential immunomodulatory and anti-fibrotic properties of NF in the present experimental setting. In this context, mechanistic data on the effects of nanofat on TGF-β1/Smad3 signaling may be particularly interesting, because this pathway markedly contributes to impaired wound healing after radiation therapy^13^. Moreover, the wound healing process was only investigated throughout a period of 14 days. However, it would have been interesting to additionally examine the long-term outcome of this process by measuring fibrosis, tissue strength and scar quality. In addition, all experiments and analyses were performed in mice, which may differ to humans in terms of skin physiology, immunology and response to ionizing radiation^35–37^. Hence, additional clinical trials should confirm these findings in chronic and non-healing wounds of patients with irradiated soft tissues. This should be easily feasible considering the fact that NF is already well established in clinical practice and can be rapidly generated without the need for neither complex nor expensive procedures and devices. Moreover, because NF is a mechanically processed autologous fat derivative, there are no major regulatory hurdles or safety requirements for its use in clinical studies, as is also the case for PRP.

## Materials and methods

### Animals

This study was approved by the local governmental animal protection committee (permission number: 06/2022; State Office for Consumer Protection, Saarbrücken, Germany) and performed according to the ARRIVE guidelines, the European legislation on the protection of animals (Directive 2010/63/EU) and the NIH Guidelines on the Care and Use of Laboratory Animals (NIH publication #85–23 Rev. 1985).

To generate a pool of PRP for the treatment of all wounds, adult male and female C57BL/6J wild-type mice (Institute for Clinical and Experimental Surgery, Saarland University, Homburg, Germany) with a mean age of 6 months were used. For NF preparation, the inguinal subcutaneous fat pads from 8 male GFP^+^ donor mice (C57BL/6-Tg (CAG-EGFP)1Osb/J; The Jackson Laboratory, Bar Harbor, ME, United States) with a mean age of 7 months and a body weight of > 30 g were excised and further processed. Moreover, 16 adult C57BL/6J wild-type mice with a mean age of 4 months and a body weight of 24 g served as recipient animals for irradiation and subsequent preparation of dorsal skinfold chambers.

### Anesthesia

To allow for the precise application of ionizing radiation, surgical procedures and microscopies, general anesthesia was induced via intraperitoneal (i.p.) injection of ketamine (100 mg/kg body weight; Ursotamin^®^; Serumwerke Bernburg, Bernburg, Germany) and xylazine (12 mg/kg body weight; Rompun^®^; Bayer, Leverkusen, Germany). Perioperative analgesia was achieved by means of a subcutaneous injection of 10 mg/kg carprofen (Rimadyl^®^; Zoetis Deutschland GmbH, Berlin, Germany).

### Localized ionizing radiation of the dorsal skinfold

**Fig. 6:**
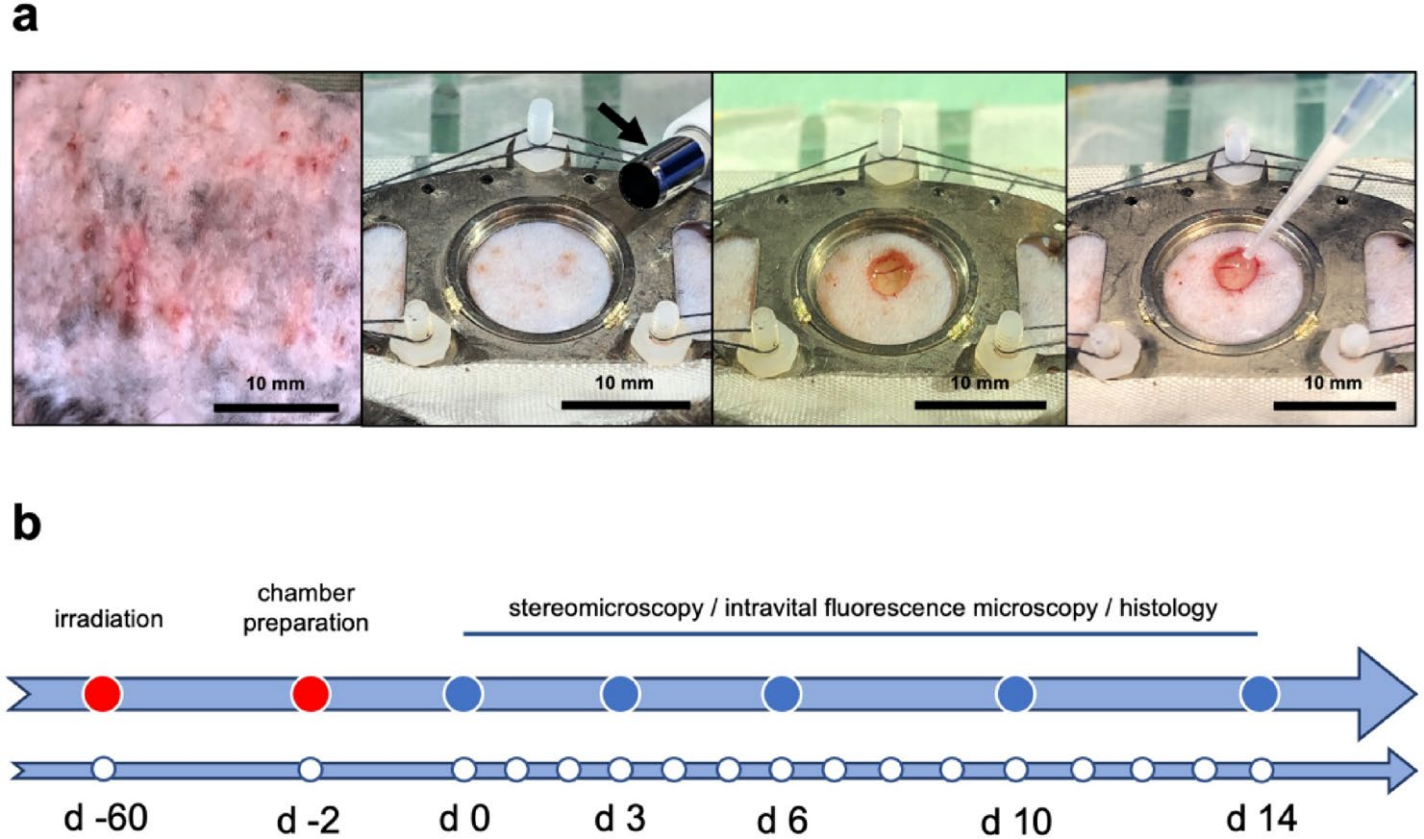
Experimental setting of the present study. (**a**) Left panel: Signs of radiodermatitis in the skin on the back of a mouse 3 weeks after localized ionizing radiation with a total dose of 20 Gy. Middle-left panel: Observation window of the dorsal skinfold chamber immediately before wound creation with a 4-mm biopsy punch (arrow). Middle-right panel: Observation window immediately after wound creation. Right panel: Filling of the wound with a combination of PRP and NF by means of a pipette. (**b**) Timeline of the in vivo experiments according to the detailed description in the materials and method section.

Mice were anesthetized and their backs were shaved and completely depilated using hair removal cream (asid med depilation cream; Asid Bonz GmbH, Herrenberg, Germany). Subsequently, their dorsal skinfold was fixed with holding sutures to a Plexiglas stage and irradiated with a total dose of 20 Gy by means of a linear accelerator (TrueBeam; Varian Medical Systems, Palo Alto, CA, USA), as previously described in detail^38^. The animals were then allowed to recover for 2 months prior to the preparation of dorsal skinfold chambers. For this purpose, they were housed in groups at a room temperature of 22–24° and a 12-h day-night cycle with free access to pellet chow (Altromin, Lage, Germany) and water. Their health status and body weight were regularly assessed. As expected, the isolated high dose irradiation of dorsal skinfolds induced typical signs of radiodermatitis (Fig. 6a).

### Generation of PRP and nanofat

PRP and NF were generated according to standard procedures, as previously described^21^. Briefly, for the generation of PRP, 3 anesthetized donor mice were fixed on a heating pad, and a median laparotomy was performed with subsequent puncture of the vena cava inferior for blood extraction (~ 1 mL per animal). The pooled harvested blood from the 3 donor mice was collected in a lithium heparin monovette (Sarstedt, Nümbrecht, Germany) and centrifuged for 15 min at 110 x g to separate the PRP from the erythrocyte pellet and platelet-poor plasma. The resulting pooled PRP was stored at -20 °C until further use for all in vivo experiments.

To generate NF, the inguinal fat pads of 8 GFP^+^ donor mice were harvested, washed in 0.9% NaCl solution and cut in smaller samples (1 × 1 × 1 mm) using a tissue chopper (McIlwain Tissue Chopper, CLE Co. Ltd., Gomshall, UK). These samples were then mechanically shuffled between two syringes using three female-to-female Luer lock connectors exhibiting decreasing internal diameters of 2.4, 1.4 and 1.2 mm and 30 passes per connector. Thereafter, the suspension was filtered through a 500-µm pore filter to remove any larger residual tissue aggregates^21^.

### Dorsal skinfold chamber model of wound healing

Wound healing was analyzed in dorsal skinfold chambers^21^. For this purpose, the previously irradiated mice were anesthetized and their back was again shaved and chemically depilated, as described above. Subsequently, the animals were equipped with dorsal skinfold chambers (Irola Industriekomponenten GmbH & Co. KG, Schonach, Germany). A detailed description of the different chamber preparation steps can be found in a previous paper by Laschke and Menger^39^. Of note, throughout the in vivo experiments, the health status of the animals was continuously monitored according to a scoring system considering (1) general condition, (2) behaviour, (3) hunching score, (4) chamber (tissue viability, infection, device displacement, suture and screw integrity) and (5) body weight. Based on this scoring system, none of the mice reached the exclusion threshold, indicating that the experiments were well tolerated by the animals.

After 2 days of recovery, the mice were anesthetized again and a full-thickness skin wound was created in the central region of the chamber observation window by means of a 4 mm biopsy punch (GSK Consumer Healthcare, GMDT, Clocherane, Youghal Road, Dungarvan, Co. Waterford, Ireland) and microscissors (Fine Science Tools GmbH, Heidelberg, Germany) (Fig. 6a). The animals were then randomly assigned to two different groups, and their wounds were either filled with a mixture of 3 µL phosphate-buffered saline (PBS) and 5 µL PRP (PRP-treated group; *n* = 8) or with 3 µL freshly generated NF and 5 µL PRP (PRP + NF-treated group; *n* = 8) (Fig. 6a). The group size of *n* = 8 animals per group was chosen as in a previous study performed in the identical model^21^. In this previous study, this group size has been proven to provide statistically valid data, while reducing the number of required animals to a minimum according to the 3R principle. Inside the wound, the PRP was activated with 2 µL thrombin (10 U/mL dissolved in 10% CaCl_2_; Sigma-Aldrich, Taufkirchen, Germany) to ensure the formation of a stable gel. This enabled the fixation of the liquid NF inside the skin defect before the chamber observation window was closed with a cover glass and a removable snap ring.

### Stereomicroscopy

In vivo stereomicroscopic analyses of the wounds were performed on days 0 (day of wound creation), 3, 6, 10 and 14 (Fig. 6b), as previously described in detail^21^. Briefly, the anesthetized animals were placed horizontally on a Plexiglas platform under a stereomicroscope (Leica M651, Wetzlar, Germany) and images were captured using a DVD system. The wound area (in % of day 0) was quantitatively assessed by means of the computer-assisted analysis system CapImage (version 8.10.1; Zeintl, Heidelberg, Germany).

### Intravital fluorescence microscopy

Directly after the stereomicroscopic analysis of the wounds, their vascularization was additionally investigated by means of intravital fluorescence microscopy^21^. For this purpose, 100 µL of the blood plasma marker 5% FITC-labeled dextran (150,000 Da; Sigma-Aldrich) was injected into the retrobulbar venous plexus of the anesthetized mice. The animals were then placed under a fluorescence epi-illumination microscope (Zeiss Axiocam 702 mono; Zeiss, Oberkochen, Germany). Image acquisition was achieved using the ZEISS ZEN software (version 3.7; Zeiss) in 6 ROIs inside the wounds. Morphological parameters of the wound microcirculation (perfused ROIs, in %; functional microvessel density, in cm/cm^2^) and microhemodynamic parameters of individual microvessels inside the wounds (diameter, in µm; RBC velocity, in µm/s; shear rate, in s^− 1^; blood flow, in pL/s) were assessed by means of CapImage^20^. After the last microscopy, the animals were sacrificed via cervical dislocation and tissue specimens were harvested and prepared for further histological and immunohistochemical analyses.

### Histology and immunohistochemistry

Harvested tissue samples were fixed in formaldehyde, embedded in paraffin and cut into 3 μm-thick serial sections. The sections displaying the largest cross-sectional diameter of the wounds were identified and stained with HE. Additional sections were stained with antibodies against Col I (1:250; Abcam, Cambridge, UK), Col III (1:100; Proteintech, Rosemont, IL, USA), CD86 (1:100; Cell Signaling, Danvers, MA, USA) and CD163 (1:100; Proteintech) for light microscopy and against CD31 (1:100; dianova GmbH, Hamburg, Germany), LYVE-1 (1:200; Abcam) and GFP (1:100; Rockland, Limerik, PA, USA) for fluorescence microscopy. A horseradish peroxidase-labeled goat-anti-rabbit IgG antibody (1:200; dianova GmbH), a goat-anti-rat IgG-Alexa555 antibody (1:100; Molecular Probes, Eugene, OR, USA) and a goat-anti-rabbit IgG-Alexa555 antibody (1:200; Molecular Probes) were used as secondary antibodies. 3-Amino-9-ethylcarbazole (Abcam, Cambridge, UK) was used as chromogen. Hoechst 33342 (2 µg/mL; Sigma Aldrich) served for the staining of cell nuclei on immunofluorescence sections. The assessment of histological or immunohistochemical parameters was performed in a blinded manner by means of a BX53 microscope and the imaging software cellSens Dimension (version 1.11, Olympus, Hamburg, Germany), as previously described in detail^21,40^.

### Statistical analysis

After testing data for normal distribution and equal variance, an unpaired Student’s t-test (parametric data) or a Mann-Whitney rank sum test (non-parametric data) were used to assess differences between the two groups (GraphPad Prism version 10.1.2; GraphPad Software, San Diego, CA, USA). All values were expressed as mean ± standard error of the mean (SEM). In addition, confidence intervals were calculated for all assessed parameters (Supplementary Tab. S1). Statistical significance was defined for the value of *p* < 0.05.

## Supplementary Information

Below is the link to the electronic supplementary material.

Supplementary Material 1

## Data Availability

All relevant data are included in the manuscript. Any additional request can be directed to the corresponding author.
