# Supplementary material for: Nanofat promotes wound healing in skin following exposure to ionizing radiation
Source: Sci Rep. 2025 Aug 29;15:31918. doi: 10.1038/s41598-025-17961-8 (PMC12397309; doi:10.1038/s41598-025-17961-8)
Supplement: Supplementary file 1 — Supplementary Material 1 [file 41598_2025_17961_MOESM1_ESM.docx]

**Supplementary Table S1**. Comprehensive summary of all quantitative parameters assessed in the present study for wounds filled with platelet-rich plasma (PRP, control) or PRP with nanofat (PRP+NF). For each parameter, the table shows the mean, standard error of the mean (SEM), 95% confidence interval (CI; lower and upper limits) and the p-value of the intergroup comparison.

|  | **PRP (control)** | | | | **PRP+NF** | | | |  |
| --- | --- | --- | --- | --- | --- | --- | --- | --- | --- |
| **Parameter** | **Mean** | **SEM** | **CI95 low** | **CI 95high** | **Mean** | **SEM** | **CI95 low** | **CI95 high** | **p-value** |
| **Body weight (1st part) [g]** |  |  |  |  |  |  |  |  |  |
| d-60 | 25.9 | 0.3 | 25.2 | 26.6 | 24.8 | 0.8 | 23.1 | 26.6 | 0.23 |
| d-59 | 25.3 | 0.3 | 24.5 | 26.1 | 24.4 | 0.7 | 22.6 | 26.1 | 0.28 |
| d-58 | 25.4 | 0.4 | 24.4 | 26.4 | 24.6 | 0.7 | 22.9 | 26.3 | 0.32 |
| d-57 | 26.0 | 0.5 | 24.9 | 27.2 | 25.0 | 0.7 | 23.3 | 26.7 | 0.27 |
| d-56 | 26.2 | 0.6 | 24.9 | 27.6 | 25.5 | 0.6 | 24.0 | 27.0 | 0.40 |
| d-55 | 26.8 | 0.6 | 25.4 | 28.2 | 26.2 | 0.8 | 24.3 | 28.2 | 0.56 |
| d-54 | 26.2 | 0.6 | 24.9 | 27.6 | 26.2 | 0.8 | 24.3 | 28.1 | 0.94 |
| d-53 | 25.7 | 0.5 | 24.6 | 26.8 | 26.4 | 0.7 | 24.8 | 28.0 | 0.40 |
| d-52 | 26.4 | 0.6 | 24.9 | 27.8 | 26.5 | 0.8 | 24.7 | 28.3 | 0.89 |
| d-51 | 27.2 | 0.6 | 25.7 | 28.7 | 26.9 | 0.8 | 25.0 | 28.8 | 0.80 |
| d-50 | 27.4 | 0.8 | 25.5 | 29.2 | 27.0 | 0.8 | 25.1 | 28.9 | 0.75 |
| d-49 | 27.5 | 0.6 | 26.2 | 28.9 | 27.0 | 0.8 | 25.2 | 28.9 | 0.62 |
| d-48 | 26.8 | 0.6 | 25.5 | 28.2 | 27.0 | 0.8 | 25.1 | 28.9 | 0.88 |
| d-47 | 26.6 | 0.5 | 25.4 | 27.8 | 26.6 | 0.8 | 24.8 | 28.5 | 0.98 |
| d-46 | 26.6 | 0.5 | 25.4 | 27.8 | 26.7 | 0.8 | 24.7 | 28.6 | 0.91 |
| d-44 | 27.3 | 0.5 | 26.1 | 28.4 | 27.4 | 0.9 | 25.3 | 29.5 | 0.90 |
| d-42 | 26.9 | 0.5 | 25.7 | 28.1 | 27.0 | 0.8 | 25.1 | 29.0 | 0.86 |
| d-40 | 26.6 | 0.6 | 25.2 | 28.0 | 26.7 | 0.8 | 25.0 | 28.5 | 0.87 |
| d-38 | 26.4 | 0.7 | 24.7 | 28.0 | 26.9 | 0.7 | 25.2 | 28.7 | 0.56 |
| d-35 | 26.9 | 0.7 | 25.3 | 28.4 | 27.0 | 0.7 | 25.5 | 28.6 | 0.86 |
| d-32 | 27.0 | 0.8 | 25.2 | 28.8 | 26.7 | 0.6 | 25.3 | 28.0 | 0.76 |
| d-29 | 26.8 | 0.7 | 25.1 | 28.5 | 27.5 | 0.6 | 26.0 | 29.0 | 0.47 |
| d-26 | 27.9 | 0.7 | 26.4 | 29.5 | 27.5 | 0.5 | 26.3 | 28.8 | 0.64 |
| d-23 | 28.1 | 0.6 | 26.7 | 29.6 | 27.8 | 0.5 | 26.6 | 29.0 | 0.68 |
| d-20 | 28.0 | 0.6 | 26.6 | 29.5 | 28.5 | 0.6 | 27.1 | 29.9 | 0.57 |
| d-17 | 29.0 | 0.6 | 27.5 | 30.4 | 28.4 | 0.6 | 26.9 | 29.9 | 0.53 |
| d-14 | 28.9 | 0.7 | 27.3 | 30.4 | 28.4 | 0.5 | 27.1 | 29.6 | 0.58 |
| d-11 | 29.1 | 0.7 | 27.6 | 30.7 | 28.6 | 0.5 | 27.5 | 29.6 | 0.47 |
| d-8 | 29.4 | 0.8 | 27.6 | 31.3 | 28.5 | 0.6 | 27.2 | 29.8 | 0.36 |
| d-4 | 29.3 | 0.9 | 27.3 | 31.4 | 28.7 | 0.6 | 27.3 | 30.0 | 0.52 |
| **Body weight (2nd part) [g]** |  |  |  |  |  |  |  |  |  |
| d-2 | 29.2 | 0.9 | 27.2 | 31.2 | 28.8 | 0.6 | 27.4 | 30.2 | 0.68 |
| d0 | 28.1 | 0.8 | 26.2 | 30.0 | 28.6 | 0.8 | 26.8 | 30.4 | 0.68 |
| d3 | 27.8 | 0.9 | 25.6 | 30.0 | 28.1 | 0.9 | 26.0 | 30.3 | 0.83 |
| d6 | 28.1 | 0.9 | 25.9 | 30.3 | 28.4 | 0.9 | 26.2 | 30.6 | 0.80 |
| d10 | 28.1 | 1.2 | 25.3 | 30.8 | 28.8 | 0.9 | 26.6 | 30.9 | 0.64 |
| d14 | 28.4 | 1.2 | 25.6 | 31.3 | 28.9 | 1.1 | 26.2 | 31.5 | 0.81 |
| **Wound area [% of day 0]** |  |  |  |  |  |  |  |  |  |
| d0 | 100.0 | 0.0 | 100.0 | 100.0 | 100.0 | 0.0 | 100.0 | 100.0 | - |
| d3 | 101.7 | 2.0 | 97.0 | 106.4 | 96.7 | 4.1 | 87.1 | 106.4 | 0.30 |
| d6 | 89.5 | 5.1 | 77.5 | 101.5 | 89.6 | 5.7 | 76.1 | 103.0 | 0.99 |
| d10 | 64.5 | 7.0 | 48.0 | 81.0 | 54.9 | 7.0 | 38.3 | 71.6 | 0.35 |
| d14 | 52.5 | 6.4 | 37.3 | 67.6 | 30.4 | 5.8 | 16.7 | 44.2 | 0.02 |
| **Perfused ROIs [%]** |  |  |  |  |  |  |  |  |  |
| d0 | 0.0 | 0.0 | 0.0 | 0.0 | 0.0 | 0.0 | 0.0 | 0.0 | - |
| d3 | 0.0 | 0.0 | 0.0 | 0.0 | 0.0 | 0.0 | 0.0 | 0.0 | - |
| d6 | 14.6 | 8.0 | -4.3 | 33.5 | 22.9 | 7.0 | 6.4 | 39.5 | 0.45 |
| d10 | 52.1 | 12.0 | 23.8 | 80.4 | 87.5 | 6.1 | 73.1 | 101.9 | 0.02 |
| d14 | 97.9 | 2.1 | 93.0 | 102.8 | 100.0 | 0.0 | 100.0 | 100.0 | 0.35 |
| **Functional microvessel density [cm/cm²]** |  |  |  |  |  |  |  |  |  |
| d0 | 0.0 | 0.0 | 0.0 | 0.0 | 0.0 | 0.0 | 0.0 | 0.0 | - |
| d3 | 0.0 | 0.0 | 0.0 | 0.0 | 1.6 | 1.6 | -2.1 | 5.3 | 0.35 |
| d6 | 4.0 | 2.3 | -1.3 | 9.4 | 17.2 | 6.3 | 2.2 | 32.2 | 0.04 |
| d10 | 33.7 | 10.5 | 8.9 | 58.6 | 62.1 | 10.0 | 38.5 | 85.7 | 0.07 |
| d14 | 81.0 | 8.8 | 60.3 | 101.8 | 114.7 | 12.6 | 84.9 | 144.5 | 0.04 |
| **Diameter [µm]** |  |  |  |  |  |  |  |  |  |
| d0 | - | - | - | - | - | - | - | - | - |
| d3 | - | - | - | - | 17.4 | - | - | - | - |
| d6 | 19.2 | 1.7 | 15.2 | 23.1 | 18.0 | 0.4 | 17.0 | 19.0 | 0.55 |
| d10 | 18.4 | 1.0 | 16.0 | 20.9 | 16.7 | 0.8 | 14.8 | 18.6 | 0.22 |
| d14 | 16.1 | 0.6 | 14.7 | 17.6 | 15.5 | 0.5 | 14.3 | 16.7 | 0.46 |
| **Centerline RBC velocity [µm/s]** |  |  |  |  |  |  |  |  |  |
| d0 | - | - | - | - | - | - | - | - | - |
| d3 | - | - | - | - | 74.7 | - | - | - | - |
| d6 | 69.2 | 9.8 | 46.2 | 92.3 | 90.7 | 12.9 | 60.1 | 121.3 | 0.22 |
| d10 | 83.1 | 5.3 | 70.6 | 95.6 | 103.3 | 17.6 | 61.7 | 144.8 | 0.31 |
| d14 | 123.9 | 19.0 | 79.1 | 168.8 | 112.4 | 19.4 | 66.6 | 158.3 | 0.68 |
| **Shear rate [s^-1^]** |  |  |  |  |  |  |  |  |  |
| d0 | - | - | - | - | - | - | - | - | - |
| d3 | - | - | - | - | 34.9 | - | - | - | - |
| d6 | 32.2 | 5.8 | 18.4 | 46.0 | 40.9 | 5.8 | 27.2 | 54.6 | 0.32 |
| d10 | 39.9 | 3.9 | 30.7 | 49.1 | 53.3 | 10.8 | 27.8 | 78.8 | 0.28 |
| d14 | 66.2 | 11.9 | 38.1 | 94.4 | 63.7 | 13.2 | 32.6 | 94.9 | 0.89 |
| **Volumetric blood flow [pL/s]** |  |  |  |  |  |  |  |  |  |
| d0 | - | - | - | - | - | - | - | - | - |
| d3 | - | - | - | - | 11.1 | - | - | - | - |
| d6 | 11.3 | 1.0 | 8.9 | 13.7 | 16.2 | 2.8 | 9.7 | 22.7 | 0.14 |
| d10 | 13.7 | 1.6 | 10.0 | 17.4 | 13.6 | 1.8 | 9.4 | 17.8 | 0.98 |
| d14 | 15.7 | 1.8 | 11.4 | 20.0 | 12.5 | 1.5 | 8.9 | 16.1 | 0.21 |
| **Epithelialization [%]** | 76.1 | 7.2 | 59.0 | 93.1 | 88.0 | 3.6 | 79.6 | 96.5 | 0.16 |
| **Granulation tissue [%]** | 63.5 | 5.0 | 51.7 | 75.2 | 76.8 | 3.9 | 67.6 | 85.9 | 0.06 |
| **Cellular density [mm^-2^]** | 2332.0 | 347.4 | 1510.6 | 3153.4 | 3007.9 | 391.9 | 2081.3 | 3934.5 | 0.22 |
| **Total Col I ratio [wound/skin]** | 0.5 | 0.1 | 0.3 | 0.7 | 0.4 | 0.1 | 0.2 | 0.5 | 0.42 |
| **Total Col III ratio [wound/skin]** | 0.5 | 0.1 | 0.3 | 0.8 | 0.7 | 0.1 | 0.4 | 0.9 | 0.35 |
| **Microvessel density [mm^-2^]** | 161.5 | 25.5 | 101.2 | 221.7 | 266.1 | 55.4 | 135.2 | 397.0 | 0.11 |
| **Lymph vessel density [mm^-2^]** | 21.3 | 6.2 | 6.7 | 35.9 | 39.1 | 10.2 | 14.9 | 63.3 | 0.16 |
| **M1 macrophages [mm^-2^]** | 114.8 | 15.5 | 78.1 | 151.5 | 87.1 | 9.4 | 64.8 | 109.4 | 0.15 |
| **M2 macrophages [mm^-2^]** | 1129.6 | 114.1 | 859.8 | 1399.5 | 1424.0 | 187.9 | 980.3 | 1868.7 | 0.21 |
| **M2/M1 macrophage ratio** | 11.0 | 1.5 | 7.3 | 14.6 | 17.8 | 2.7 | 11.4 | 24.2 | 0.05 |
